# Necrosis, and then stress induced necrosis-like cell death, but not apoptosis, should be the preferred cell death mode for chemotherapy: clearance of a few misconceptions

**DOI:** 10.18632/oncoscience.61

**Published:** 2014-07-03

**Authors:** Ju Zhang, Xiaomin Lou, Longyu Jin, Rongjia Zhou, Siqi Liu, Ningzhi Xu, D. Joshua Liao

**Affiliations:** ^1^ CAS Key Laboratory of Genome Sciences and Information, Beijing Institute of Genomics, Chinese Academy of Sciences, Beijing, P.R. China; ^2^ Hormel Institute, University of Minnesota, Austin, MN, USA; ^3^ Department of Genetics & Center for Developmental Biology, College of Life Sciences, Wuhan University, Wuhan, P. R. China; ^4^ Laboratory of Cell and Molecular Biology, Cancer Institute, Academy of Medical Science, Beijing, P.R. China

**Keywords:** Apoptosis, Cancer therapy, Carcinogenesis, Evolution, Hyperthermia

## Abstract

Cell death overarches carcinogenesis and is a center of cancer researches, especially therapy studies. There have been many nomenclatures on cell death, but only three cell death modes are genuine, i.e. apoptosis, necrosis and stress-induced cell death (SICD). Like apoptosis, SICD is programmed. Like necrosis, SICD is a pathological event and may trigger regeneration and scar formation. Therefore, SICD has subtypes of stress-induced apoptosis-like cell death (SIaLCD) and stress-induced necrosis-like cell death (SInLCD). Whereas apoptosis removes redundant but healthy cells, SICD removes useful but ill or damaged cells. Many studies on cell death involve cancer tissues that resemble parasites in the host patients, which is a complicated system as it involves immune clearance of the alien cancer cells by the host. Cancer resembles an evolutionarily lower-level organism having a weaker apoptosis potential and poorer DNA repair mechanisms. Hence, targeting apoptosis for cancer therapy, i.e. killing via SIaLCD, will be less efficacious and more toxic. On the other hand, necrosis of cancer cells releases cellular debris and components to stimulate immune function, thus counteracting therapy-caused immune suppression and making necrosis better than SIaLCD for chemo drug development.

For over three centuries, i.e. since 1665 when the word “necrosis” first emerged, most pathologists and biologists had been familiar with only this single form of cell death, although a few compeers had noticed and described some quite different morphologic traits of dead cells, observations which are now considered to be the first descriptions of programmed cell death [1]. However, ever since 1972 when Kerr et al created the word “apoptosis” to describe some distinctive morphologic traits of cell death in tumor tissues [2], the number of concepts on cell death, each associated with a some sort of mechanism, has been increasing rapidly in the literature [1]. The following are some of these nomenclatures: necrosis, regulated necrosis, programmed necrosis, apopnecrosis, netosis, necroptosis, apoptosis, extrinsic apoptosis, intrinsic apoptosis, oncosis, anoikis, mitotic catastrophe, excitotoxicity, Wallerian degeneration, paraptosis, parthanatos, pyroptosis, pyronecrosis, entosis, cornification, and autophagic cell death [3-6]. Many of these nomenclatures may have overlap in the demise mechanisms they describe, but probably very few cell death experts can tell all the details of these, and other unmentioned, cellular death modes. In this essay, we describe our musings on cell death in animals and on its relevance to cancer therapy, which is a continuation of previous descriptions of cellular death [1,7].

## Stress-induced cell death (SICD) is programmed

Most tissues or organs in an evolutionarily high-level animal have a routine cell turnover, with newly generated cells replacing the dead ones. When a cell in these tissues or organs encounters endogenous stress such as spontaneous DNA mutation, or an exogenous stress such as radiation or a toxic agent that causes DNA damage, the cell will first arrest itself, usually at the G1 or S phase of the cell cycle [8], so that the damaged DNA can be repaired. If the damage is irreparable, the cell will commit to a suicidal program to kill itself, so as to prevent passing genetic alteration to its progeny cells. Because the dying or dead cell may be engulfed by a macrophage or another type of scavenger cell, similar to apoptosis described before [1,7], this type of demise is defined herein as “stress-induced apoptosis-like cell death (SIaLCD)”. Because it involves another cell type as the scavenger, SIaLCD does not occur in routine cell culture systems, just like apoptosis [1,7].

The dead cells may not always be engulfed by scavenger cells, especially when the death is too massive for the clearance capacity of the available scavengers, which often occurs in irradiation or chemotherapy of a cancer. In this situation, the dead cells are likely to decompose to debris via a necrotic process and may trigger an inflammatory response in an in vivo situation, which sometimes is called ‘secondary necrosis” [9]. Therefore, this form of cell death is dubbed herein as “stress-induced necrosis-like cell death” (SInLCD). Although its early steps are programmed events, in reality SInLCD is actually difficult to distinguish from necrosis. Most published studies with a tissue have some cells dying of necrosis and others dying of SInLCD or SIaLCD, whereas most studies using cell culture systems have some cells dying of necrosis while others dying of SInLCD. Because these two or three modes of cell demise appear concomitantly in a system and it is difficult, if not impossible, to distinguish individual dying cells in a Petri dish or a tissue, usually cells dying from different modes are mixed and collectively studied, which may be a situation that is described as the “programmed necrosis”, “regulated necrosis”, “netosis”, or “necroptosis”. In other words, these *ad hoc* concepts are created to amalgamate the irreconcilable necrosis and apoptosis and are unnecessary, since SICD is a reconcilable mixture of the two.

## What are the similarities and differences among apoptosis, SICD and necrosis?

**Fig 1:**
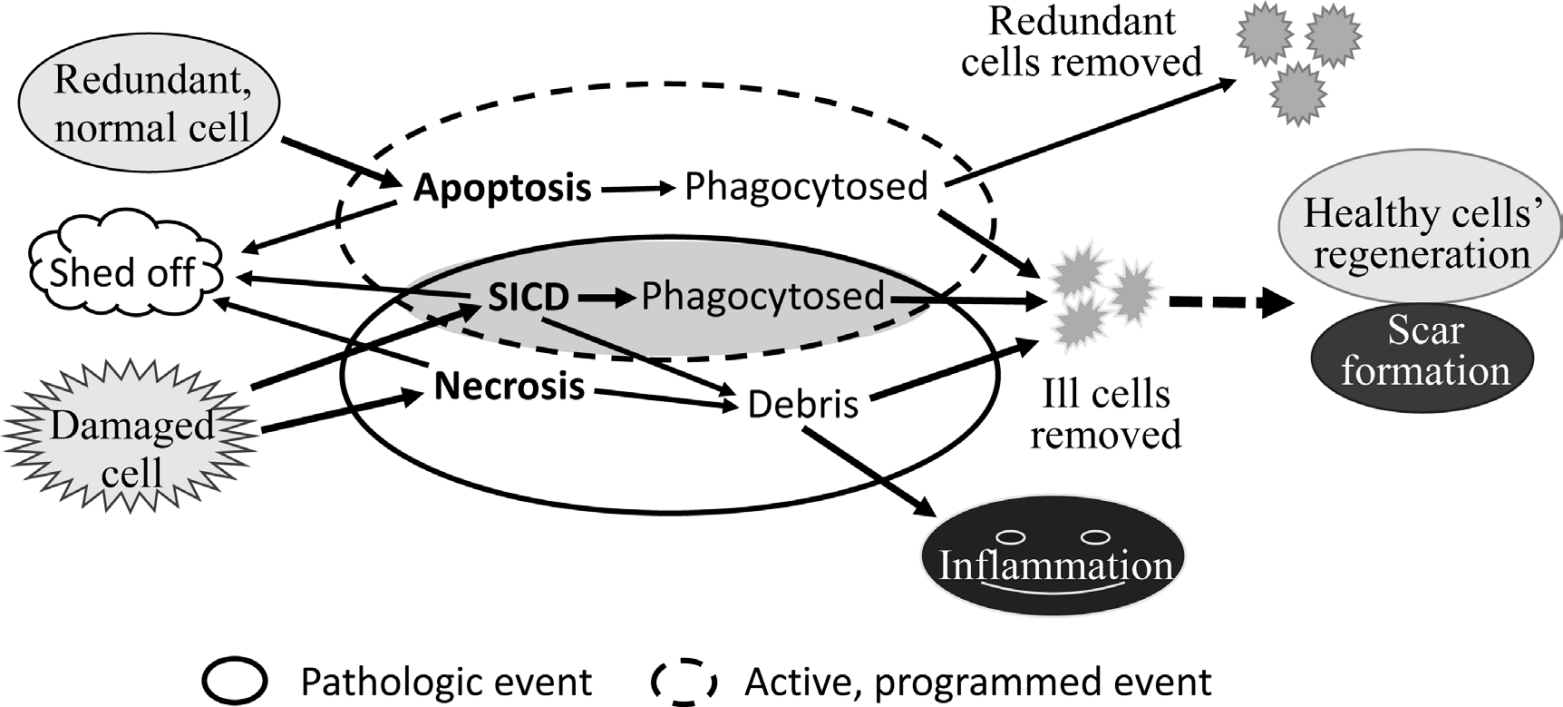
Depiction of three basic cell death modes While apoptosis is a physiological event (large circle by dashed-line), canonical necrosis is a pathological, passive event (large circle). Stressed induced cell death (SICD) manifests some apoptosis properties and some necrosis properties as indicated by the overlapping area of both circles. Other similarities and differences among the three types of cell demise are detailed in the text.

In our humble opinion, there are only three major cellular death mechanisms, i.e. apoptosis, necrosis and SICD, for most animal tissues or organs in most situations, with SICD containing two subtypes, i.e. SIaLCD and SInLCD. Some specific forms of cell death are excluded as they occur only in some specific organs or situations, such as cornification in the skin and anoikis in cell culture. If the dead cells are located at external or luminal surfaces, they will slough from the skin or will shed into the lumen and then be excreted out of the body as a component of feces, urine, milk, sweat, phlegm, saliva, etc (Fig. 1). These cells can die from any of the three mechanisms but will not be discussed herein because the dead cells are quickly discarded from the body and thus do not affect the physiology of the animal.

Like apoptosis, SIaLCD and the early steps of SInLCD are programmed events. In the case of SIaLCD, the dying or dead cells will be engulfed by macrophages or other scavenger cells, resembling the process in apoptosis. However, these active suicidal events occur because the ill or damaged cells give their allegiance to the tissue or the animal as the whole and thus are willing to die for the purpose of maintaining the genome integrity of the cell community, i.e. to prevent passing DNA mutations to progeny cells. Moreover, the cell death will trigger regeneration of healthy sibling cells to restore the physiological cell number, and thus full function, of the organ or tissue. If the cell loss is massive, connective tissue cells, mainly fibroblasts, may enter into the region and proliferate to fill in the space, a process in pathology textbooks termed “ wound healing and scar formation” (Fig. 1), exemplified by the liver cirrhosis caused by alcohol or chronic hepatitis B virus infection. In sharp contrast, apoptosis has developed during evolution for the purpose of removing “no-longer useful” cells from an animal and therefore is not followed by regeneration of the healthy sibling cells and scar formation by connective tissue. For example, mammary gland cells in a lactating dam are no longer useful and will die of apoptosis after the pups wean, which is not followed by regeneration and scar formation. In other words, the cells removed via apoptosis can be perfectly healthy although they are useless, whereas the cells removed via SICD are ill or damaged although they are useful. SICD, either SIaLCD or SInLCD, is a pathologic event occurring in an abnormal situation, such as when there is an irreparable DNA damage, whereas apoptosis is a physiologic event in a normal situation, although where the demarcation between “normal” and ‘abnormal” lies is often not as clear as black-and-white. To use an analogy, if a person has food to eat when he feels hungry or has water to drink when he feels thirsty, the temporary hungry or thirsty status is still normal. However, a too-long hungry or thirsty period without food or water can be a pathological situation, although how long a period is “too long” is hard to define as well.

Both necrosis and SICD are due to stress. SICD resembles canonical necrosis not only in the nature of pathology but also in the ensuing regeneration and possible scar formation that have been described before [1,7] (Fig 1). However, SICD is a programmed suicide and may be triggered by an endogenous factor such as spontaneous DNA mutation, whereas necrosis is a passive homicide wherein the cells do not want to die but are killed by physical (e.g. radiation), chemical (e.g. carcinogens or chemotherapeutic drugs) or biological (e.g. bacteria, viruses or even insufficient oxygen) factors outside the cell. Also as explained previously [1,7], the physiological, programmed apoptosis that removes redundant but probably healthy cells and thus does not trigger regeneration and scar formation contrasts sharply to the pathological, passive necrosis in which useful cells are killed by exogenous factors, followed by regeneration and possible scar formation (Fig 1).

In routine cell culture systems, there is no canonically defined apoptosis because 1) the cells are not motivated to prevent inflammation and the ensuing scar formation and 2) there is no second cell type available as the scavengers [1,7]. Accordingly, there is no SIaLCD in cell cultures. In other words, cells in a Petri dish can die of only necrosis or SInLCD, and likely both occur concomitantly.

## Tumors resemble low-level organisms

All cells in an animal will die after they reach their life spans, but the life spans of different cells in the same animal are quite different. For example, neurons and heart muscle cells, which no longer divide after childhood and thus do not develop sporadic cancer, have much longer life spans than most other cell types in a normal situation. Sporadic tumorigenesis, a process for the development of a benign or a malignant sporadic tumor, starts with immortalization of one somatic cell [10], which likely involves alteration in genomic DNA. Here, “immortality” does not mean that the immortalized cell itself is immortal, but, instead, it means that the cellular death program has been reprogrammed to allow the cell to divide for limitless rounds, much over the Hayflick limit that is about 50 generations in vitro [11-13]. In our rumination, this reprogramming revokes the legality of immortalized cell lines in exploration of apoptosis mechanisms, because the identified “mechanisms” cannot reflect the true apoptosis programs in the cells of a living animal [1,7]. Reiterated, it is inappropriate to use cell lines, whose death program has been reprogrammed, for much of the exploration of apoptosis pathways.

Immortalization actually converts a cell to a new species of organism, as the cell can sustain the new, slightly altered genome by limitless rounds of cell division. In other words, the immortalized cell has autonomy, which can be restated as loss of its allegiance to the host tissue or the animal. This loss of allegiance while gaining autonomy is a course of progressive evolution. Many tumor promoting agents, such as many chemical carcinogens, promote malignant transformation actually by utilizing the cell’s allegiance to the organ: These agents not only kill normal cells via necrosis or SICD but also inhibit proliferation, so-called “mitoinhibition” [8,14], of the remaining normal cells, which imposes regeneration pressure onto those cells that, due to whatever reason, have previously been initiated to be resistant to the mitoinhibition. These preexisting initiated cells with the “resistant phenotype”, which are small in number (probably just one in a spontaneous situation), are those that eventually evolve to be immortalized, as depicted in detail before [8,14]. Once immortalized, the cell’s main interest is to establish and sustain its own family as an independent organism, with little concern on the host. Actually, like parasites, it will do whatever damage to the host that is needed for it to maintain a better life, which is why a tumor kills the patient and why we need to study it.

**Fig. 2:**
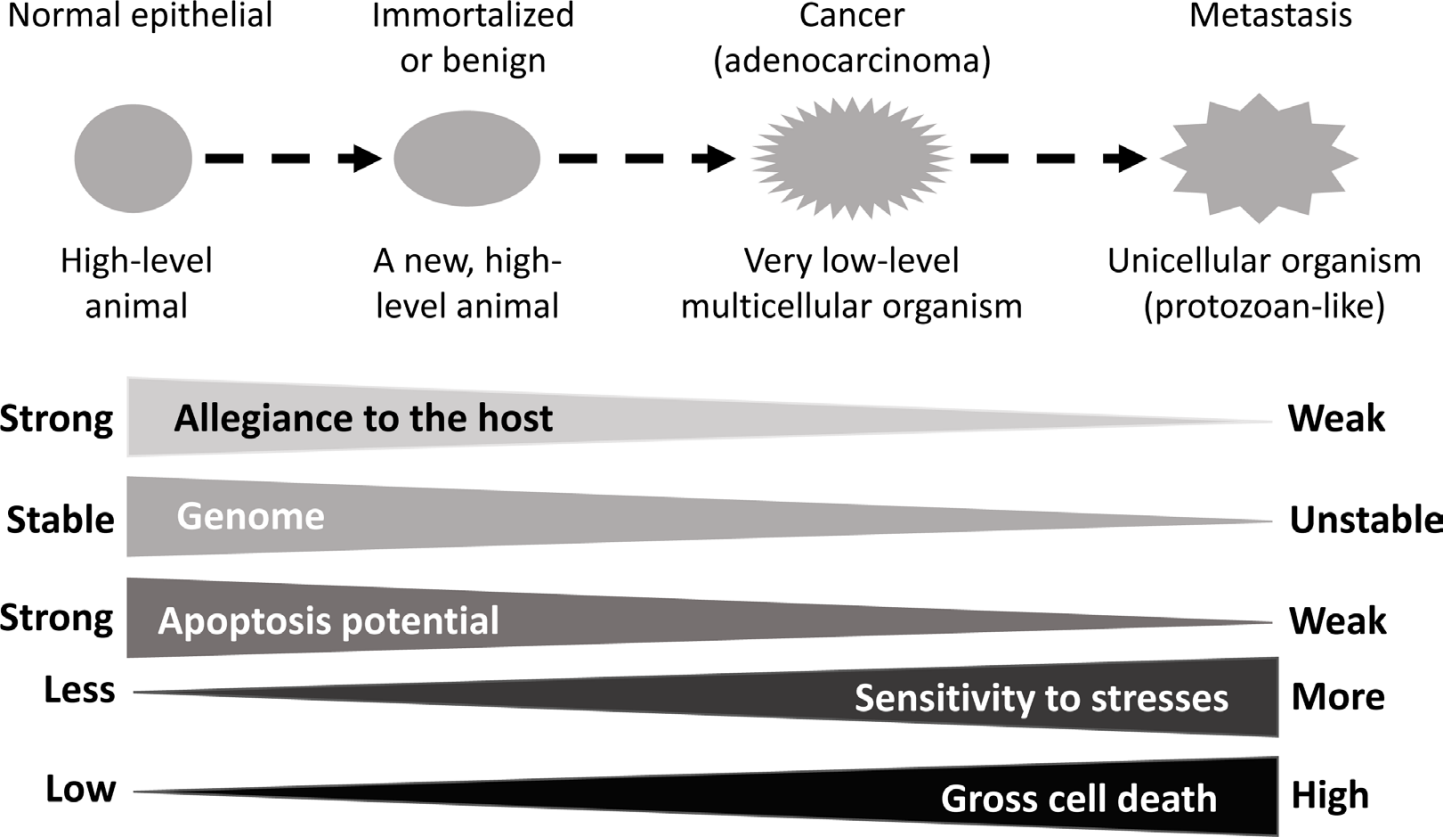
Illustration of stepwise carcinogenesis as an atavistic process of a somatic cell in animals, with epithelial carcinogenesis as an example Immortalization renders a somatic cell immortal and autonomous. Autonomy means that the cell has lost or partly lost its allegiance to the host tissue or the animal and concerns itself mainly with sustaining its altered genome, but not with sustaining the host. Therefore, the immortalized cell actually incepts a new organism. Continuing on the carcinogenic process, sometimes to a status of benign tumor as an intermediate, this new organism loses more differentiation features and thus becomes more and more malignant and genomically unstable. At the molecular level, this progressive atavism is featured by continuously emerging mutations as happenstances and ensuing selections of genotypes characterized by functional gain of survival-sustaining oncogenes and functional loss of differentiation-sustaining tumor suppressor genes. Some cancer cells eventually break away from the tumor mass to be individuals disseminating into the surrounding stroma, somewhat like unicellular protozoans. The autonomous nature of cancer cells also provides them with decreasing apoptosis potential but increasing sensitivity to various forms of stress, manifested as a higher cell death toll compared with the host tissue or organ. All these traits put the organism at an even lower position in the life tree.

In the cases of solid malignant tumors, the immortalized cell continues on an atavistic process, i.e. a reverse evolution in which the cell gradually evolves towards intermediate organisms of lower and lower levels on the life tree, manifested as e.g. ductal or glandular cancer masses and then as invasive cells that break away from the tumor mass to be individuals disseminating in the surrounding stromal tissue, somewhat similar to unicellular protozoans (Fig. 2). Actually, the course from “breaking away” to colonizing at the new body sites somewhat resembles sporulation and subsequent germination of bacteria that require signals from other bacterial cells [15]. During this atavism, cells gradually lose differentiation both in morphology and in function, compared with their normal counterparts in the same tissue or organ, but not with the very first founder cell that was immortalized, because currently it is still unclear whether sporadic cancers are derived from differentiated cells that are de-differentiated or from blastocytes (tissue-specific stem cells) that stop differentiation. Probably, both types occur but in different cases [16]. Mechanistically, these cellular changes are largely attributed to continuously emerging mutations as happenstances and to ensuing selections, from these mutations, of genotypes characterized by functional gain of survival-sustaining oncogenes and functional loss of differentiation-sustaining tumor suppressor genes (Fig. 2). Because cancers lose differentiation and morphologically resemble embryonic cells, pathology textbooks traditionally borrow a set of terms from embryology such as “undifferentiated” or “poorly differentiated” to describe cancer. As expected, the gene expression profile is also very similar between highly aggressive carcinomas and normal stem cells [17,18]. In the eyes of pathologists, the more malignant the cancer cells are, the more they resemble stem cells in embryonic tissues, generally speaking. Conversely, cells of some benign tumors may be morphologically indistinguishable from the normal cells in the host tissue. Actually, due to this reason, so far there still is a lack of chemo agents that can preferentially kill benign tumor cells, such as uterine myoma cells, without equally damaging their normal counterparts.

## In a tumor living like a parasite, cell death is not an apoptosis of the host patient

The notion that carcinogenesis is an atavism yielding tumors as new, lower-level organisms explains why a family of materials excreted from microorganisms, usually coined with the suffix of “-mycin” such as geldanamycin, bleomycin, mitomycins, actinomycins and neomycins, not only kill other microorganisms nearby, as such “-mycins” are supposed to do, but also have a good efficacy window for cancer therapy while sparing normal cells that are higher at the life tree. A difference is that a tumor is a “macro-organism” visible to the naked eye and parasitizing the host patient. The notion that a tumor resembles a parasite is of importance in tumor biology as it says that death of some tumor cells occurs within the “parasite” and thus is not an apoptosis of the host. Moreover, not only because cancer is a different organism but also because this organism is evolutionarily lower than the host animal, cells of the cancer no longer care about inflammatory damage they may cause to the host, whereas prevention of such inflammation and the ensuing tissue damage is the motivation for apoptosis, i.e. is why animals have evolved to equip themselves with apoptosis [1,7]. Therefore, cancer no longer requires a sophisticated apoptotic mechanism. In other words, with their loyalty to the tissue or organ, cells in animals may undergo apoptosis if the body requires them to do so, but this is no longer relevant to cancer cells that are autonomous and no longer give their allegiance to the host.

As expounded in detail previously [1,7], apoptosis requires a neat coordination between the dying cell and a macrophage (or another scavenger cell) that will engulf the dying or dead cell. Although a tumor is a lower-level organism, it may still retain some apoptosis mechanism, just like most lower-level animals or metazoans, but it is evolutionarily preliminary and may not involve engulfment of the apoptotic cell by a scavenger that migrates from a distant site along blood or lymphatic vessels, similar to apoptosis in *Caenorhabditis elegans* that lacks these circulation systems [7]. The more malignant the cell, the lower it is in the life tree, and thus it has a lesser apoptosis potential and a simpler mechanism, with metastatic cells that resemble unicellular protozoans at the lower end. This is one reason why metastatic lesions are more refractory than the primary tumor to such chemo drugs that kill via apoptosis-involved SIaLCD mechanism, as to be discussed later.

While a cancer sickens the host animal, just like a parasite, the animal may mobilize macrophages and other scavenger cells to clean it up, if the animal realizes that it is being “parasitized” or, more correctly, if the tumor “leaks” out some immunizing signals for the host to detect it. What many peers may not realize is that engulfment of tumor cells by macrophages of the host origin is not an apoptosis of either the parasitic organism or the host animal (such as a human), simply because the prey and the predator belong to two different organisms. Rather, it is something similar to an immune action of the host for clearance of such aliens as bacterial cells, which is one reason why cancer cell death often manifests features of necrosis, besides other cell death modes. Actually, there are indeed several types of parasitic cancer in nature that spread by direct tumor cell transmission, and one of them (canine transmissible venereal tumor) can regress spontaneously due to immune clearance by the host [19-21]. Probably, such *ad hoc* concepts as “netosis”, “necroptosis” and “programmed or regulated necrosis” are created partly because peers do not separate apoptosis of the parasitic tumor from the host’s immune clearance of aliens.

## Potentials of mitoinhibition and apoptosis cause a tradeoff between adverse effects and therapeutic efficacy

**Fig. 3:**
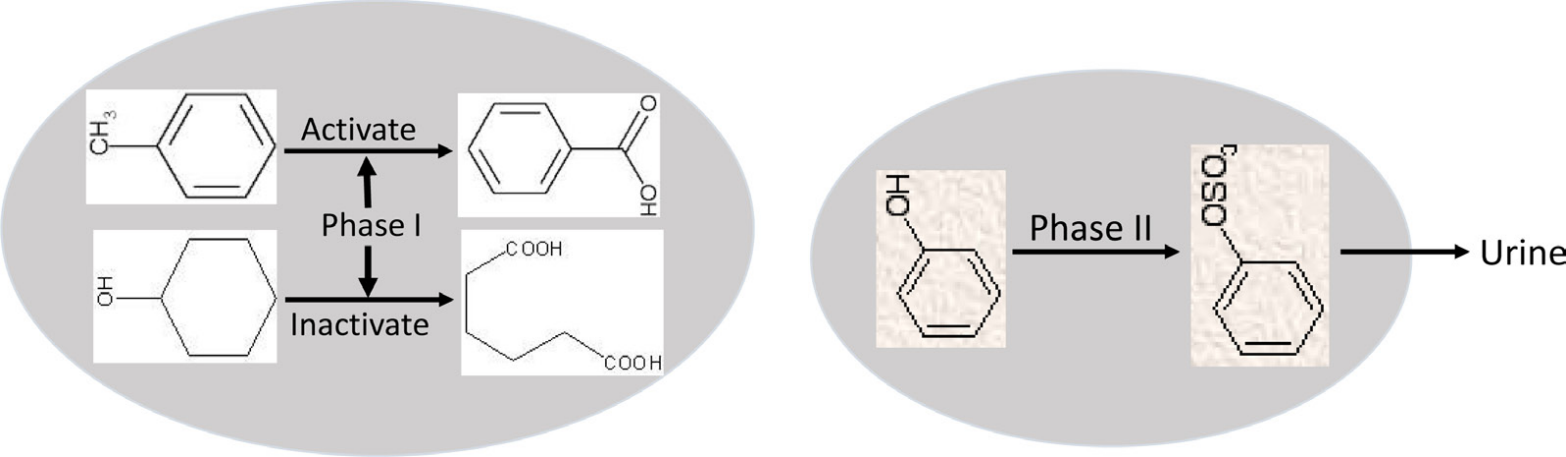
Illustration of effects of phase I and phase II drug-metabolic enzymes Phase I enzymes catalyze oxidation of chemicals such as a carcinogen or a chemo drug, which may increase or decrease the chemical’s toxicity, depending on the chemicals to be oxidized. Phase II enzymes conjugate compounds, such as phenol, to a chemical group, which usually increases their excretion from the body via such routes as urine or feces, one example being the excretion of sulfate phenol in the urine.

Xenobiotics and some endogenous metabolic intermediates of cells usually are oxidized first (phase I), mostly by cytochrome P450 enzymes, and then are conjugated (phase II) to a chemical group to be hydrophilic and easier to excrete out of the body [22], exemplified by the sulfation of phenol and the ensuing excretion of the phenol sulfate from the cell to urine (Fig. 3). While the levels of phase II metabolic enzymes are usually elevated in immortalized cells and in their derived cancer cells, the changes of phase I enzymes are complex, because oxidation of some chemicals such as some carcinogens leads to detoxification or inactivation whereas oxidation of some others can activate them and thus cause harm to the cell [23-26]. The increased or decreased phase I enzymes and the usually increased phase II enzymes in cancer constitute an important mechanism for the resistant phenotype, as this metabolic profile leads to a quicker inactivation and clearance of some chemo agents [27,28]. Also due to this difference in the metabolic profile, when attacked by a xenobiotic, normal cells usually arrest proliferation (i.e. “mitoinhibition”) to minimize genomic and cellular damage, whereas cancer cells are relatively less mitoinhibited and continue proliferation, manifesting chemo-resistance.

Like radiation, many chemo drugs act mainly by causing DNA damage [29,30]. Actually, other types of cancer therapy more or less hit DNA and cripple the DNA repair mechanism as well [31,32]. For instance, hyperthermia therapy has such effects [33,34], which is not surprising as it has been known for 90 years that most placental mammals have evolved the scrotum to keep testes 2-7 °C lower than the body temperature to prevent sperm from being mutated [35]. Although they are normally in a hypoxic situation, due to the anatomy and the histology of the testes [36], spermatogenic cells are probably the fastest proliferating ones in the bodies of these animals, unlike their female counterparts in the ovaries. Increase in temperature, so called heat stress, effectively kills spermatogenic cells [37-39]. Actually, even at normal temperature (37 °C), spermatogenesis ceases. Patients whose testes fail to descend from the abdomen to the scrotum are not only infertile but also at a higher risk of developing testicular cancer. On the other hand, testicular cancer, including its metastases to other organs with a normal temperature of 37 °C, is among the most curable malignancies [40], which is probably related in part to the property that male germ cells becomes fragile at 37 °C. Hence, a lower temperature has evolved to prevent mutations from occurring in highly proliferating cells [41], especially under a hypoxic situation.

Cancer cells have lost or partially lost response to DNA damage, and in turn loss of checkpoint controls [30], due to mutations such as in p53, ATM, PETEN, BRCA1, etc. Moreover, cancer cells often have an impaired DNA repair mechanism as well [30]. For these reasons, damaged DNA will not be repaired as efficiently as in normal cells, and genotoxic treatments cause more severe DNA damage in cancer than in normal cells. Many of these cancer cells can no longer survive and die of SCID because the damage leads to the loss of too many genes needed to sustain their life. Actually, even in the absence of therapy, most cancers manifest a much larger number of dead cells than the corresponding normal tissue. A phenomenon familiar to all pathologists who read cancer slides is that, of those still-alive cells, many are not as healthy as others and thus have a weaker growth ability, which, however, does not entitle those with a stronger ability to grow, including in soft agar and xenograft model [42], to be “cancer stem cells”.

**Fig. 4:**
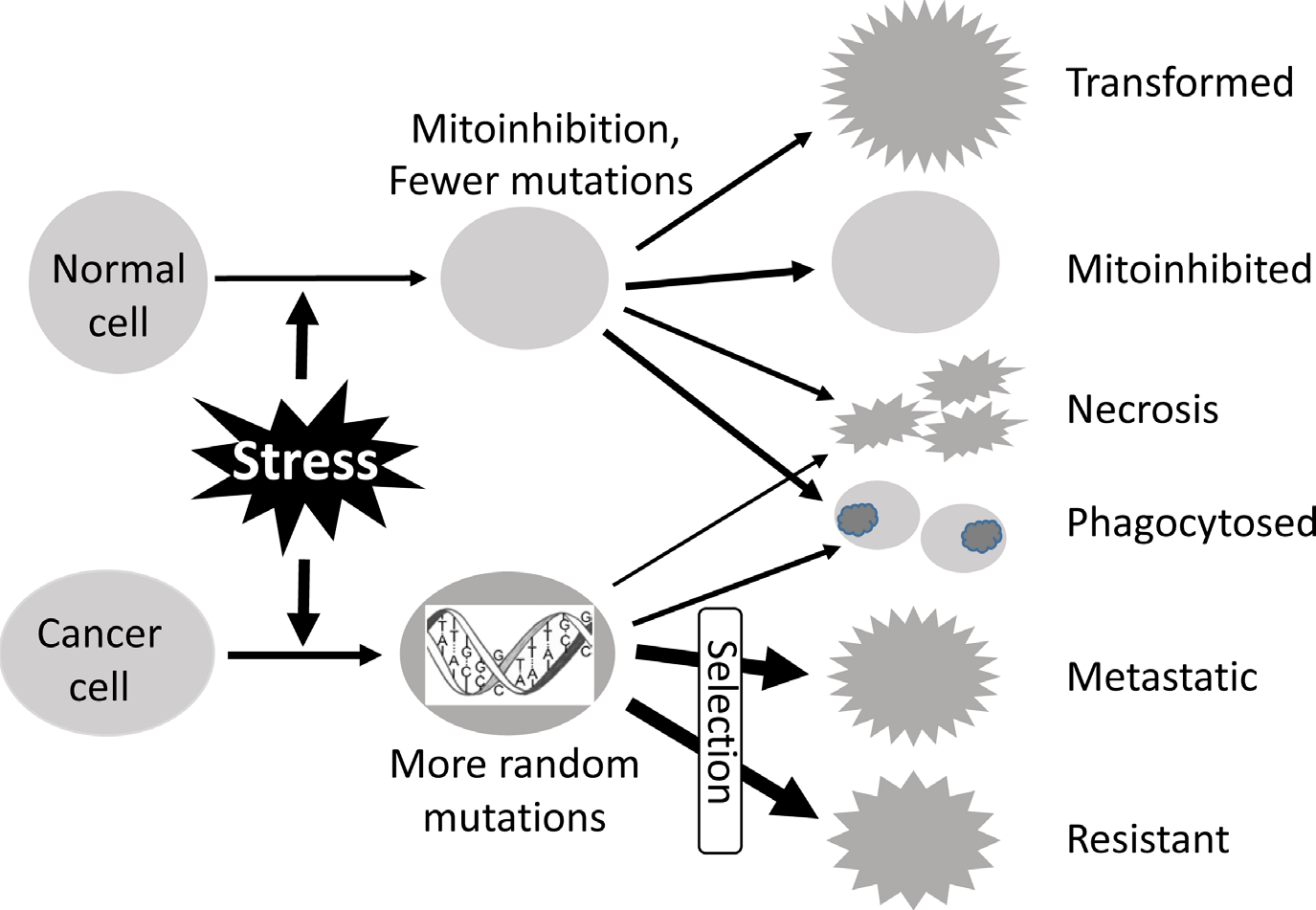
The same intensity of genotoxic stress (radiation, chemotherapy, virus, etc) causes more severe DNA damage in cancer cells than in normal cells This is not only because normal cells will arrest their proliferation (mitoinhibition) and thus are less damaged but also because normal cells can repair damaged DNA more efficiently, owing to their intact repair mechanisms. However, also because normal cells have an intact DNA repair mechanism and a stronger apoptosis potential, the same extent of DNA damage causes a higher death toll in normal cells, compared with cancer cells. The cells die via not only necrosis and SInLCD, both producing cellular debris to stimulate inflammation, but also via SIaLCD that involves phagocytosis of cell bodies. This tradeoff at least attenuates, if it does not fully eliminate, the therapeutic index of genototoxic stress. The weaker repair mechanisms and lesser apoptosis potential also lead to more severe DNA damage, which in turn provides live cancer cells with more resources to undergo Darwinian selection for those clones that are resistant to the stress and probably also that are capable of metastasizing to and colonizing in distant body sites where the microenvironment is less stressful. On the other hand, some normal cells that bear a few critical mutations may be transformed to malignancy, which is usually manifested as a second primary tumor.

Causing more severe genomic damage to cancer cells is a reason why genotoxic agents can preferentially kill cancer cells (Fig. 4). However, the fact has another aspect: also because normal cells can better detect DNA damage, more easily cause checkpoint arrest for DNA repair, and have a stronger apoptosis potential, all being part of the mechanism behind mitoinhibition [14], the same extent of DNA damage not only kills more normal cells but also causes more severe mitoinhibition of still-alive normal cells. Moreover, because highly proliferating bone marrow cells, skin epidermal cells, gastrointestinal (GI) mucosal epithelia, etc, are growth-arrested, patients manifest common side-effects including lower blood cell counts, skin itch, GI symptoms (nausea, vomiting and diarrhea), etc. From the standpoint of history, the idea of targeting fast-growing cells for the development of chemo drugs was actually originated from these side-effects, especially the suppression of the immune system, leading neutropenia to being a standard clinical testament of dose-limiting toxicity. Mustard gas was reported in 1919 to potently suppress bone marrow [43,44]. This phenomenon led mustine to being the first chemical studied and clinically tried for cancer therapy in 1942 [45,46], while it was concurrently proven during World War II in hundreds of people who developed profound lymphoid and myeloid suppression after an accidental exposure to Allied forces’ own mustard gas during a German air raid in Bari of Italy. In his report on this once classified information of these victims, Alexander theorized that since mustard gas ceased the division of certain types of somatic cells whose nature was to divide fast, it could also potentially be used in helping to suppress the division of certain types of cancerous cells [“History of cancer chemotherapy” in Wikipedia, also [47]]. This rationale formally started the chemotherapeutic strategy of targeting fast-growing cells.

The same intensity of genotoxicity causes more severe DNA damage in cancer cells that have a weaker DNA damage response and an impaired DNA repair mechanism, whereas the same extent of DNA damage causes a more severe growth arrest and a higher cell death toll in normal cells (Fig. 4). This tradeoff occurs with a genotoxic type of stress and is mechanistically attributed to the apoptosis and mitoinhibition potentials of the cell. Therefore, we surmise that this tradeoff may be less evident with a general, i.e. cytotoxic, type of stress and thus make this type of anticancer drugs better than the genotoxic type.

Restoration of cell cycle checkpoint for DNA repair, typically by restoration of p53, is a common chemo strategy to kill some types of cancer cells, especially those that have lost functional p53 [30,48]. However, those normal cells whose p53 is also raised will die of SIaLCD as well. Conversely, inhibition of cell cycle checkpoints is also a common strategy used to kill some other types of cancer cells via induction of mitotic catastrophe [30;48]. Similarly, those normal cells whose checkpoints are also inhibited will accumulate many mutations; as the consequence, they will either die of loss of too many genes or develop a second primary tumor. Moreover, if a cancer contains these two types of cells, which is highly likely, either of the two strategies may kill some cancer cells but in the meantime may promote the growth or survival of some others.

## The order of chemo strategy is better as necrosis, SInLCD and then SIaLCD

**Fig 5:**
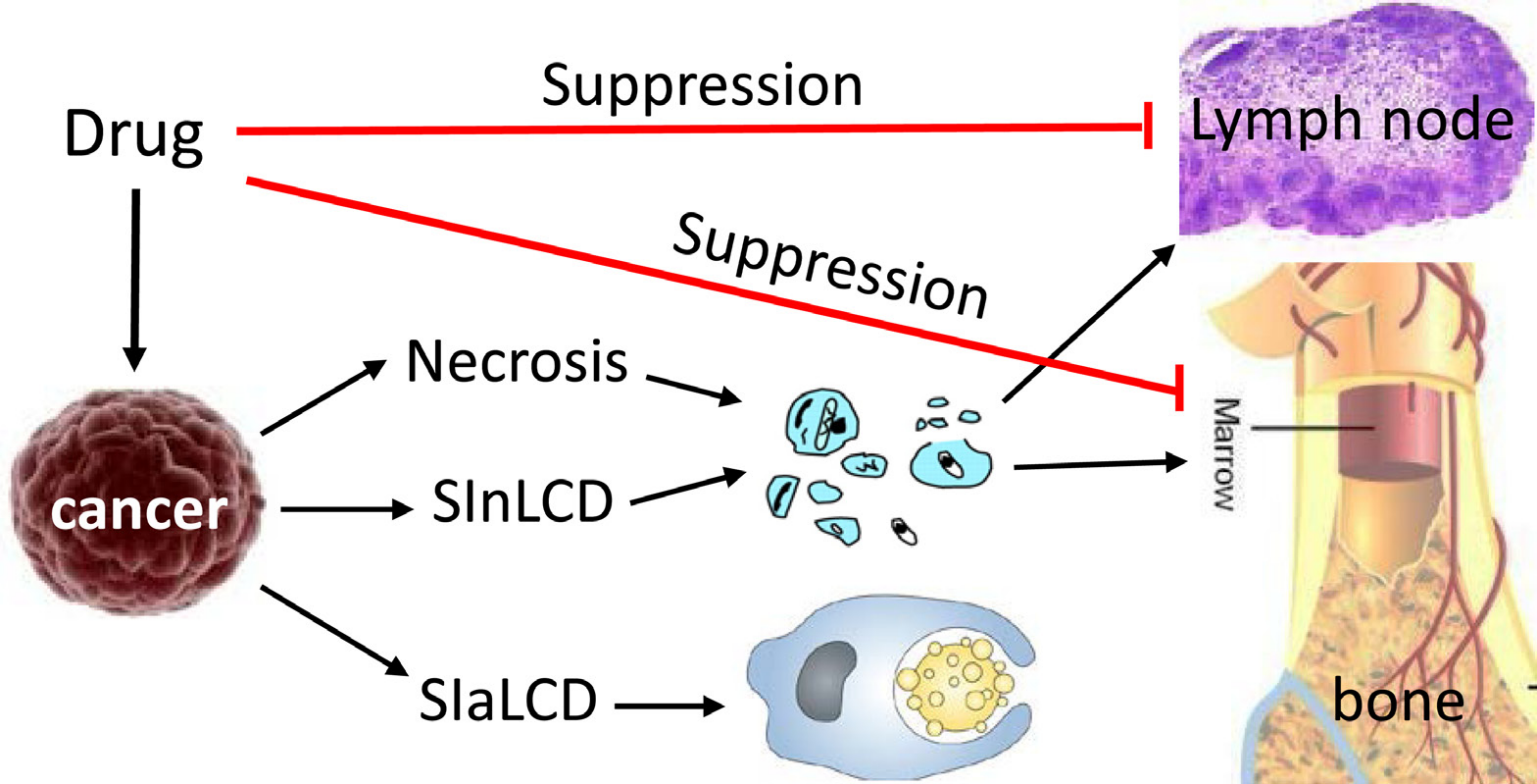
A tradeoff between necrosis- or SInLCD-induced immune stimulation and drug-induced immune suppression Chemo drugs often kill cancer cells via necrosis, resulting in release of cell debris and various immunogenic components to stimulate immune functions and inflammatory response of the patient, which in turn will elicit cancer cell specific killing. If the drugs as a stress kill cancer cells via SInLCD, there is also release of the immunogenic materials and the ensuing cancer cell specific killing. However, if the drugs kill via a SIaLCD mechanism, which is the main goal of most published studies, the dead cells will be scavenged promptly via phagocytosis by mainly macrophages, without releasing any immunogenic components and thus without the ensuing immune killing. On the other hand, chemo drugs often suppress immune functions via a mélange of mechanisms, including mitoinhibition of bone marrow cells and different lymph cells (such as dendritic cells and natural killer cells). Therefore, killing via necrosis and SInLCD will trigger a secondary immune attack on cancer cells and is thus better than killing via SIaLCD.

Cancer cells in a patient may die from one of the three mechanisms, i.e. killed 1) by itself (i.e. suicidal apoptosis), 2) by an agent outside of the cell such as a drug, and 3) by the host patient, although in our “parasite-host” analogy the host is also an outside factor and can be integrated into the second category. The first mechanism is intrinsic, i.e. within the cancer cells. As explained hereinabove, because cancer cells still retain some loyalty to the cancer as an independent organism, they still retain some apoptosis potential. Although it still remains enigmatic to us whether cancer as an organism may have some redundant cells to be removed via apoptosis, it certainly has ill or damaged cells to be removed via SIaLCD, such as those having spontaneous DNA damage that is irreparable. Although a tumor mass does not have its own macrophages, the dying or dead cancer cell can be engulfed by a neighboring tumor cell, as described by Kerr et al in their seminal work in which in the word “apoptosis” was created [2]. This is, or resembles, an authentic apoptosis without releasing any cellular debris to stimulate the host animal to respond with inflammation. The second mechanism is actually a SICD, as the cancer cells are killed by an exogenous stress, including a physical (i.e. radiation), chemical (i.e. chemo agent) or biological (i.e. viral vaccine) factor. When the dead cells are few in number, the cell bodies may still be engulfed by neighboring tumor cells as described above. However, because cancer cells have only a poor phagocytosis capacity, in reality most cancer cells dead from the first and second mechanisms, especially during irradiation and chemotherapy, will decompose to cell debris, resembling a necrosis or SInLCD. Moreover, a drug can commit the cells to necrosis via general cytotoxicity, just like many man-made or natural toxins, which is probably the most dominant mechanism within the second category (Fig. 5). The patient’ body will also attack the parasitic tumor using a mélange of humoral and cell-mediated immune mechanisms, which is put into the third category because the death is not initiated by an apoptosis potential. Some of the tumor cells may be engulfed by macrophages of host origin, which resembles SIaLCD but is not an apoptosis of either the cancer or the patient because the prey and the predator belong to different organisms.

Chemo drugs not only kill cancer cells but also, unfortunately, often suppress immune functions via many mechanisms [49;50], including mitoinhibition of bone marrow and various lymph cells such as dendritic cells and natural killer cells (Fig. 5) [51,52]. Necrosis and SInLCD of cancer cells result in cell debris and various components that are immunogenic and can stimulate immune functions [53-57], including the activation of dendritic cells and natural killer cells to elicit cancer-cell specific killing. SIaLCD does not have this benefit, as it does not release any immunogenic components. Therefore, necrosis and SInLCD are better than SIaLCD as strategies for chemotherapy (Fig. 5). Moreover, since SInLCD is initiated by an apoptosis potential and thus results in the abovementioned tradeoff between therapy efficacy and adverse effects (Fig. 4), its efficacy is not as good as necrosis.

A therapy is a killer or a stress causer to cancer cells and thus can induce either necrosis or SICD (either SIaLCD or SInLCD), but not apoptosis. Some similarities between SIaLCD and apoptosis depicted in figure 1 have resulted in a widespread and long-standing misconception that cancer therapies kill via apoptosis. Cancer as a quasi-alien somewhat resembling a parasite in the host animal involves an immune clearance of the alien cells by the host, which makes this misconception established even more easily. This misconception unfortunately makes SIaLCD the most popular choice as the strategy to develop chemo drugs. As described above, SIaLCD-based therapies have two weaknesses, i.e. a tradeoff between efficacy and adversity (Fig. 4) and a lack of release of immunogenic components to stimulate immune function (Fig. 5). Therefore, the order of therapeutic strategy, with regard to the cell death mode, is necrosis, SInLCD, and then SIaLCD.

Because cancer resembles an individual organism and its cells retain some allegiance to the cancer as the whole, loss of cancer cells from whatever mechanisms may trigger regeneration. Therefore, it remains possible that surgical removal or killing by other therapies may trigger, by imposing a regeneration pressure, the remaining cancer cells to grow at an even faster rate [58].

## Targeted therapy should be reevaluated as it has weakness

Mutations occur in a huge number of genes in cancer cells [59], with some genes mutated at multiple sites. Some mutations occur more often, dubbed “hot spots”, to provide growth- or survival-advantage, and thus are commonly considered as the best targets for so-called “targeted therapies”. However, although targeted therapies have been touted as “magic bullets” in past decades [60], so far they have not yet provided patients with appreciably longer survival than the traditional chemo drugs like 5-fluorouracil (since 1950s) and cisplatin (since 1960s) that are less specific in targets [61,62]. Most targeted therapies currently used clinically, generally speaking, provide only 9-14 months of response before resistance occurs [61-63], which is modest or even meagre but with exorbitant costs, for some of the newer ones estimated to be about $200,000 to $300,000 per quality-adjusted life year produced in the US [64,65]. In fact, Americans are more likely to die of cancer today (225.4 per 100,000) than in 1950 (195.4 per 100,000) [49], and many patients actually die of therapy-caused health deterioration, but not of cancer *per se* [66-68], suggesting that prevention and treatment of cancer have not been improved as substantially as in other diseases.

Most, if not all, cancer cells have lost at least one DNA-damage-repair mechanism, making the cells completely dependent on an alternative repair pathway. Compromising this alternative can induce cancer-cell specific death while sparing normal cells in which this pathway is functionally redundant [29]. This is the concept of synthetic lethality, an advanced version of targeted therapy [69,70], which is supported by the finding that some mutations are mutually exclusive [71,72]. For instance, tumor cells that bear BRCA1 or BRCA2 mutation are exquisitely sensitive to a PARP1 inhibitor, olaparib [73-75]. Whereas simultaneously targeting all oncogenic pathways to kill all cancer cells is not realistic, a good combination of magic bullets to simultaneously target two or several survival pathways may be doable and be synthetically lethal to many cancer cells [76,77]. However, we surmise that any combination may, in the meantime, also synthetically spur the growth or survival of some other cancer cells [8]. In addition, targeted therapy may trigger resistance much more easily than cytotoxic therapy, because cancer cells can establish alternative survival pathways easily [62,63]. Unfortunately, research on these potential adversities may not be rewarding to peers and thus is still lacking. Moreover, cancer cells are in a continuous atavism with mutations and ensuing selections of resistant cell clones constantly going on. A longer duration of treatment will give the cancer a longer time to accumulate more mutations in individual cells and select more formidable clones, which requires us to reconsider targeted therapy.

## General cytotoxic therapy, such as hyperthermia, should gain more attention as it has merits

Most currently used chemo agents mainly cause DNA damage and thus have the aforementioned weakness, i.e. a tradeoff between therapeutic efficacy and the adverse effects (Fig 4). Therefore we need to shift much of our attention back onto the general approaches such as hyperthermia and controls of osmotic pressure, oxygen partial pressure, acidity-basicity (pH), and ion channel [78-85] that have been studied for a long time [80,86-91]. These approaches may still cause DNA damage indirectly as described for hyperthermia but mainly elicit general toxicity to which cancer cells are more vulnerable, due to their high acidity and other metabolic features [80,90,92] and because their heat shock protein levels are already high before treatment [62,93-95]. If lowering the temperature from 37 to 35 °C can effectively prevent DNA mutation in the testes, as aforementioned, raising it to a feverish range (e.g. 39 °C) may increase mutations and thus commit the cells to SICD. Actually, a recent study shows that increasing the ambient temperature from the routine 22-23 °C to 30-31 °C significantly decreases xenograft tumor growth in mice [86].

In 1867 Busch in Germany used a cotton-wool bandage to transmit bacteria from an erysipelas patient onto a small burn injury of a sarcoma patient, which caused tumor remission within two weeks, although the tumor regrew after the patient recovered from the erysipelas [96,97], unlike the complete remission without regrowth seen in his earlier patients with two infections [98,99]. In 1882 Fehleisen confirmed this therapy and further identified *Streptococcus pyogenes* as the causative agent of erysipelas [100]. In 1887 Bruns also cured a recurrent melanoma with erysipelas and summarized 14 reported cases with complete or stable remission [101]. During 1891-1936, Coley at New York injected a bacterial mixture of, first live but then heat-killed, gram-positive *S pyogenes* and gram-negative *Serratia marcescens* [102-104] to patients with bone- or soft-tissue sarcoma or with certain epithelial cancers [105]. Ironically, this so-called “Coley’s toxin” appears to be a better treatment than any currently used regimens since about 500 of the 1000 patients treated by Coley and others with this toxin showed significant tumor regression, according to the analyses by Coley’s daughter [106-109] and others [52,102,110,111]. Also magically, the Coley’s toxin can alleviate tumor-caused pain [105]. Likely, this bacterial mixture is not only an immunotherapy [102,112] but also works through hyperthermia, as the efficacy largely depends on whether the patients responded with a high fever [105,108]. Actually, stimulation of immune function is a significant part of the mechanism behind hyperthermia therapy [85,113-117], although it remains unknown whether this is because innate immunity also regulates thermogenesis [118,119]. In our conjecture, most mammals set 37 °C as the optimal temperature for most of their functions but a feverish temperature for the best immune function, because a massive immune defense is not routinely required. This may be a reason why infection is often associated with a fever, evolutionarily. The monthly slight elevation of basal body temperature in women, usually used to herald ovulation, is probably for enhancing the immune function to protect the egg and its fertilization and conception from infection in the uterus, since only one egg is produced each month and thus is so valuable. Retrospectively, some researchers speculate that since many patients receiving Coley’s toxin were immune-compromised by prior treatment with chemotherapy or radiation, the efficacy should be much better if the toxin is used alone [52,110,120]. Nevertheless, today’s physicians no longer have interest in Coley’s toxin since it is risky and tortures the patients with months of great discomfort [52], despite that its last use, performed in China in the late 1980s, showed a complete regression of a terminal liver cancer [121,122].

Animals sometimes drop their temperature when they are exposed to toxic chemicals [123], likely because a lower temperature can decrease the metabolic rate and toxicity. Many cancer patients also manifest hypothermia or feel “cold” during chemotherapy [124-128], possibly because the body recognizes the chemo drug as a toxin and thus lowers the temperature and the metabolic rate to minimize its “toxicity” [123]. If this inference is correct, raising the body temperature may restore the chemo efficacy. On the other hand, so often animals, including cold-blooded species like fish [129], move to a warmer area when they are ill [130]. Many studies show that increased body temperature is required for maximal survival of animals of different species after experimental infection [131-136], whereas hypothermia can decrease the resistance to post-surgical infection [137-139]. Acute febrile infection has been, anecdotally, associated with many cases of spontaneous cancer regression [49,97,140-151] and prophylaxis [120,152-157]. Hyperthermia therapy is actually an ancient medical approach used as sauna, sweat lodge or spa (hot water bath or balneotherpay) in many countries [158,159]; it alone or in combination with chemotherapy or irradiation is effective for some cancers [33,89,90,114,142,160,161]. No wonder Parmenides, an ancient Greek philosopher (about 540-480 B.C.), said that “give me the power to induce fever, and I cure all diseases” [97], and Hippocrates (479-377 B.C.) also said that “those who cannot be cured by medicine can be cured by surgery. Those who cannot be cured by surgery can be cured by fire (hyperthermia). Those who cannot be cured by fire, they are indeed incurable” [160,162].

While Coley used bacteria as pyrogens to incite fever in cancer patients, in Austria Julius Wanger-Jauregg successfully cured neurosyphilis, a form of tertiary syphilis, by inducing high fever with the malaria parasite, a species of protozoan, through blood transfusion from malaria patients to the syphilis victims. Although this perilous therapy killed some patients and can no longer be used, it led Wanger-Jauregg to the 1927’ Nobel Prize for Medicine [163,164]. Interestingly, Deidier already noticed in 1725 that tumors of syphilitic patients were cured more often than others and that prostitutes infected with syphilis had a lower frequency of cancer than the average population [97]. Moreover, one century ago D’Arcy Power also observed an inverse correlation of malaria to cancer, and wrote “where malaria is common, cancer is rare” [49,165].

## Treatments at the maximal dose may be perilous but have tantalizing merits

Inspired by the perilous but more-effective regimens of Busch-Fehleisen-Coley and Wanger-Janregg, we postulate, with trepidation, a better therapeutic strategy by increasing the stress intensity as high as possible, i.e. close to a shock level to run against time. Because this maximized therapy will kill more cancer cells in the least time, it does not leave the cancer with sufficient time to accumulate mutations and select formidable clones, although such maximal stress is more precarious than the conventional treatments and probably can only be carried out in hospitalized patients under intensive care. Patients have to make a choice between the potential for a longer survival with a higher risk and a routine therapy that provides only 9-14 months of response, providing that the extortionate financial outlay is not a concern. Probably, “cure me or kill me” may be the request from many patients who are in the throes of cancer. There were six deaths among the about 1,000 cases treated with Coley’s toxin a century ago [52], but we probably can do a better job now. What has been baffling us for a long time is why in 1867 Bruch could shrink a cancer simply with a cost-free cotton-wool bandage [96,98] whereas today we still have not yet improved much the prognosis with many sophisticated instruments or devices and exorbitant medicines. We may need to reconsider our therapeutic strategies and principles, such as inducing necrosis instead of apoptosis (actually SIaLCD).

The currently dominant strategy of targeting proliferating cells leaves out the dormant or stem-cell-like cancer cells that replicate only occasionally, leaving these cells with a chance to repopulate one day. This is another reason for the need of reconsideration. Many drugs designed to target cell proliferation, such as those mitosis inhibitors, have very promising efficacies in inhibiting cancer cells in culture dishes and shrinking tumors in xenograft models but do not work in patients, especially those with solid tumors [31,166,167]. A great disparity between the laboratory and the bedside is that cell- or tumor-doubling time in culture dishes or xenograft models is only several days but in human patients is several months [31,166,167]. While this disparity remains to be a conundrum, it indicates that many more cells in the culture dishes or xenograft tumors than in the patients are in the proliferating fraction and thus are targeted [31,32,166-168]. Using these agents as controls, we should question whether those that show a good clinical efficacy really act mainly by targeting proliferating cells as we assume and as our laboratory data suggest [31,32,166-168]. Also because all drugs kill a lot of cells in the xenograft tumors, many more than the clearance capacity by macrophages, in reality SInLCD or necrosis, but not SIaLCD, is always the dominant cell death mode in the xenograft models. Theoretically, we should select those slowly-growing cell lines to best mimic human cancers, but in reality many, if not most, peers do the opposite by selecting the fast-growing cell lines, so as not only to get positive data for publication and grant purposes but also to finish the studies, especially costly animal experiments, in the shortest time period. This may be a reason why many “promising” compounds cannot go beyond the phase III clinical trials.

## SUMMARY

Cell death is always a center of cancer researches, especially therapy studies that are aimed to causing cancer cell specific death. There have hitherto been many nomenclatures on cell death in the literature, likely with more to emerge continuously. However, in our humble opinion, most of them are *ad hoc* concepts whereas only three basic cell death modes are authentic, i.e. apoptosis, necrosis and SICD, with SICD containing two subtypes (SIaLCD and SInLCD). SICD and apoptosis overlap at their programing nature whereas SICD and necrosis overlap at their pathology nature and their triggers of regeneration and scar formation. Whereas apoptosis removes redundant or no-longer useful but healthy cells, SICD removes useful but ill or damaged cells. Many *ad hoc* nomenclatures such as “apopnecrosis” or “netosis” may be created not only because SICD shares some properties with necrosis and apoptosis but also because in most experimental systems SICD appears concomitantly with necrosis and/or apoptosis. Moreover, many studies on cell death are carried out using cancer tissues that resemble parasites in the host patients, which is a system that further complicates the situation as it involves immune clearance of the alien cancer cells by the host. Sporadic carcinogenesis as a progressive atavism constantly results in evolutionarily lower-level organisms that are manifested first as primary benign and malignant tumors and then as more-aggressive, e.g. metastatic or therapy-resistant, tumors. Because cancer cells have a much weaker apoptosis potential and poorer DNA repair mechanisms, targeting apoptosis for chemotherapy, i.e. killing via SIaLCD that is initiated by the cell’s own apoptosis potential, will be less effective on cancer and more toxic to normal cells. Therefore, it is a widespread and longstanding misconception that directing cancer cells to SIaLCD (which is often mistaken as apoptosis) is the best chemotherapeutic strategy. On the other hand, necrosis of cancer cells engenders cellular debris and many immune-stimulatory components to stimulate immune function, thus counteracting the immune suppression caused by chemo agents and making necrosis a better cell death mode than SInLCD for chemotherapy. Considering that most targeted-therapies currently used clinically improve the survival only modestly but with exorbitant costs, general therapies with less target specificity should be used, such as hyperthermia therapy, probably in combination with targeted therapy. Moreover, all treatments probably should be used at maximal doses under intensive care to kill cancer cells as many as possible in the least time, so that the still-alive cells are fewer and do not have sufficient time to accumulate mutations and then select more refractory clones to repopulate to more intractable tumors.
